# Long-term outcomes of cataract surgery with toric intraocular lens implantation by the type of preoperative astigmatism

**DOI:** 10.1038/s41598-022-12426-8

**Published:** 2022-05-19

**Authors:** Tetsuro Oshika, Shinichiro Nakano, Yoshifumi Fujita, Yuya Nomura, Yasushi Inoue, Hiroyasu Takehara, Kazunori Miyata, Masato Honbou, Toru Sugita, Tsutomu Kaneko

**Affiliations:** 1 Department of Ophthalmology, Faculty of Medicine, University of Tsukuba, 1-1-1 Tennoudai, Tsukuba, Ibaraki 305-8575 Japan; 2 Division of Ophthalmology, Ryugasaki Saiseikai Hospital, Ryugasaki, Ibaraki Japan; 3 Fujita Eye Clinic, Tokushima, Japan; 4 Inoue Eye Clinic, Okayama, Japan; 5 Miyata Eye Hospital, Miyazaki, Japan; 6 Sugita Eye Clinic, Tokyo, Japan

**Keywords:** Eye diseases, Lens diseases

## Abstract

Surgical outcomes of toric intraocular lens (IOL) implantation for 8 years after surgery were analyzed. Data were retrospectively collected in 176 eyes of 176 patients before and 1 month, 1, 3, 5, and 8 years after phacoemulsification and implantation of a toric IOL. Preoperative corneal and postoperative manifest astigmatism was analyzed by converting to power vector notations; horizontal/vertical (J_0_) and oblique (J_45_) astigmatism components. Toric IOL implantation significantly reduced pre-existing astigmatism by decreasing J_0_ in eyes with preoperative with-the-rule (WTR) astigmatism, increasing J_0_ in eyes with against-the-rule (ATR) astigmatism, and correcting J_45_ in eyes with oblique astigmatism. After surgery, the eyes with preoperative ATR astigmatism showed a significant ATR astigmatic shift, and J_0_ at 5 and 8 years was significantly smaller than that at 1 month postoperatively. Uncorrected distance visual acuity was also significantly worse at 5 and 8 years than at 1 month postoperatively. In eyes with WTR and oblique astigmatism, the effects of toric IOLs on astigmatism and visual acuity were sustained for 8 years. The long-term astigmatism-correcting effects did not differ among the models of toric IOL used in this study, SN6AT3–8 (Alcon Laboratories). In eyes with preoperative ATR astigmatism, astigmatism-correcting effects of toric IOLs decreased at 5 years and later postoperatively, indicating that overcorrection may be considered at the time of cataract surgery. In eyes with WTR and oblique astigmatism, the effects of toric IOLs were maintained throughout the 8-year follow-up period.

## Introduction

The efficacy and usefulness of toric intraocular lens (IOL) implantation to reduce or neutralize pre-existing corneal astigmatism at the time of cataract surgery are well addressed^1,2^. Various factors are known to help improve the surgical outcomes of toric IOLs, such as introduction of sophisticated biometry and lens power calculation formula^3–7^, advent of image-guided digital marking systems^8–11^, refinement of IOL surface finish and design to reduce misalignment after surgery^12,13^, better understanding of factors that influence postoperative rotational stability of toric IOLs^14,15^, and widespread use of surgical techniques to minimize surgically induced astigmatism. Longitudinal studies on the results of toric IOLs, however, have been confined to 1- or 2-year follow-up^16–23^, and long-term outcomes of toric IOLs have not been well studied^24^. Especially, little is known how long the astigmatism-correcting effects of toric IOLs continue or diminish over years. In addition, long-term changes in visual acuity after toric IOL implantation remain unclear. We conducted the current retrospective study to investigate the time-course of changes in the effects of toric IOLs for 8 years after surgery, in consideration of the influence of preoperative corneal astigmatism type.

## Patients and methods

### Patients

We retrospectively reviewed the medical records of patients who had undergone phacoemulsification and implantation of a toric IOL for age-related cataract at six surgical sites. Only patients who were followed up for 8 years or longer after surgery were selected. If both eyes of a patient had been operated on, the first-operated eye was included in the analyses. Toric IOLs were indicated for eyes having corneal regular astigmatism of 0.75 diopter or more. Eyes were excluded from the subjects if they had corneal diseases, retinal diseases, glaucoma, uveitis, and other diseases that influence postoperative visual acuity or refraction. Those with a history of ocular surgery other than cataract surgery were also excluded. Eyes that underwent laser capsulotomy for posterior capsule opacification were included. Eyes with significant IOL misalignment > 15 degrees were excluded from analyses. Contact lens users were not included in the subjects.

For the IOL power calculation, the axial length and curvature of corneal anterior surface were measured using the IOLMaster (Carl Zeiss Meditec GmbH). The target refraction was not necessarily emmetropia but determined according to patients' preference. Using the designated manufacturer’s online calculator program, the IOL cylinder power and alignment axis were calculated. The surgically induced astigmatism (SIA) of 0.2–0.4 D was used for calculation.

Since this was a retrospective study, surgical procedures were not standardized before surgery. In general, the following procedures were employed. Preoperatively, with the patients in an upright seated position to avoid cyclotorsion errors, the corneal limbus was marked along the principal meridians at the slit lamp. At the beginning of surgery, the target axis for toric IOL alignment was identified and manually marked with a spatula or marker on the limbus. Following cataract removal through a temporal corneal or sclerocorneal incision, a toric IOL (SN6AT3–8, Alcon Laboratories, Fort Worth, Texas, USA) was implanted in the capsular bag using an injector and rotated to the final position by aligning the reference marks on the IOL with the limbal axis marks. No suture was placed on the incision. Limbal relaxing incision or astigmatic keratotomy was not conducted. Eyes with intraoperative complications were excluded from the subjects. None of the eyes underwent secondary surgical intervention to reposition the toric IOL for the correction of axis misalignment.

The study adhered to the tenets of the Declaration of Helsinki, and the institutional review board of Tsukuba University Hospital approved the study protocol. The committee waived the requirement for patient informed consent regarding the use of their medical record data in accordance with the regulations of the Japanese Guidelines for Epidemiologic Study issued by the Japanese Government. Clinical trial registration was not required owing to the observational nature of the study. All participants were at least 18 years old.

### Data analysis

Preoperative keratometry, postoperative manifest astigmatism, and postoperative uncorrected (UDVA) and corrected distance visual acuity (CDVA) were analysed. The preoperative corneal astigmatism was categorized into three types; astigmatism in which the steeper meridian was within ± 30 degrees of the vertical axis was defined as with-the-rule (WTR), astigmatism with the steeper meridian of ± 30 degrees of the horizontal axis was classified as against-the-rule (ATR), and all others were considered to be oblique astigmatism.

Before the data were converted into power vector components, the astigmatic data from the left eye were mirrored horizontally to retain the correct nasal/temporal orientation with the right eye. The preoperative corneal and postoperative refractive astigmatism was decomposed into two components, vertical/horizontal and oblique astigmatism components, using the power vector analysis described by Thibos et al.^25^. The vertical/horizontal astigmatism component is expressed as the Jackson cross-cylinder, axes at 90 degrees and 180 degrees (J_0_), and the oblique astigmatism component as the Jackson cross-cylinder, axes at 45 degrees and 135 degrees (J_45_). A positive J_0_ represents WTR astigmatism, and a negative J_0_ indicates ATR astigmatism. A positive J_45_ shows counter-clockwise oblique astigmatism, while a negative J_45_ denotes clockwise oblique astigmatism.

### Statistical analysis

Numerical data are presented as mean ± standard deviation unless otherwise noted. For assessment of data taken at different time points, the repeated measures analysis of variance (repeated measures ANOVA) was used, followed by a post hoc test with the Bonferroni correction. Comparison of paired variables was conducted using the paired t-test. All statistical tests were 2-sided and a p-value of less than 0.05 was considered significant. Statistical analysis was performed using the SPSS software version 27 (IBM Corp, Armonk, NY).

A pre-study power calculation using a significance level of 5% (α) and a power of 80% (1-β) showed that a sample size of 31–40 would be required to detect a clinically relevant difference in astigmatism between preoperative and postoperative time points. In this calculation, a standard deviation of changes in astigmatism was assumed based on previously published studies^24,26^.

## Results

### Astigmatism

Data from 176 eyes of 176 patients were analysed. Their age at the time of cataract surgery was 72.8 ± 8.6 years old and ranged from 42 to 84 years old. Patients’ demographics are summarized in Table 1. The model of toric IOLs used was SN6AT3 in 61 eyes, SN6AT4 in 56 eyes, SN6AT5 in 45 eyes, SN6AT6 in 5 eyes, SN6AT7 in 6 eyes, and SN6AT8 in 3 eyes. The number of eyes examined at each point was 176 preoperatively, 176 at 1 month, 145 at 1 year, 160 at 3 years, 165 at 5 years, and 176 at 8 years postoperatively.

**Table 1:** Patients’ demographic.

	All eyes	Type of preoperative corneal astigmatism		
		With-the-rule	Against-the-rule	Oblique
Number of eyes	176	44	121	11
Age	72.8 ± 8.6 (42–84)	67.8 ± 8.7 (42–83)	74.7 ± 7.8 (47–82)	72.3 ± 8.8 (56–84)
Left/right eyes	85/91	27/17	55/66	3/8
Preoperative J_0_ (diopter)	− 0.193 ± 0.767 (− 2.486 to 2.362)	0.791 ± 0.519 (0.100 to 2.362)	− 0.567 ± 0.510 (− 2.486 to − 0.010)	− 0.010 ± 0.170 (− 0.309 to − 0.276)
Preoperative J_45_ (diopter)	− 0.004 ± 0.301 (− 1.108 to 0.846)	0.019 ± 0.349 (− 0.719 to 0.846)	0.028 ± 0.215 (− 0.880 to 0.684)	− 0.514 ± 0.408 (− 1.108 to − 0.062)

Mean ± standard deviation (range).

**Figure 1:**
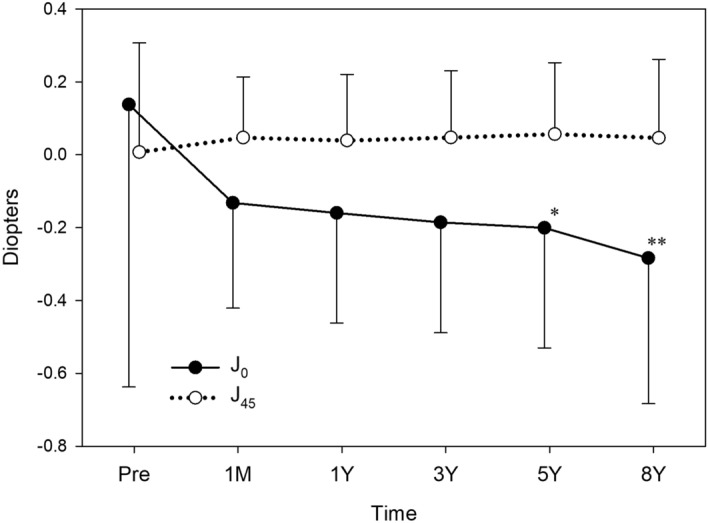
Time course of changes in astigmatism. Toric IOL implantation significantly decreased J_0_ (*p* = 0.002), but not J_45_ (*p* = 0.902). When postoperative values from 1 month to 8 years were compared, statistically significant changes were found in J_0_ (*p* < 0.001) but not in J_45_ (*p* = 0.743). The J_0_ at 5 years (*p = 0.008) and 8 years (**p < 0.001) were significantly smaller than J_0_ at 1 month postoperatively.

The time course of changes in J_0_ and J_45_ are shown in Fig. 1. The mean J_0_ significantly decreased after surgery (*p* = 0.002), while mean J_45_ did not show significant changes (*p* = 0.902). All postoperative J_0_ values from 1 month to 8 years were significantly smaller than the preoperative J_0_ value (*p* < 0.001).

**Figure 2:**
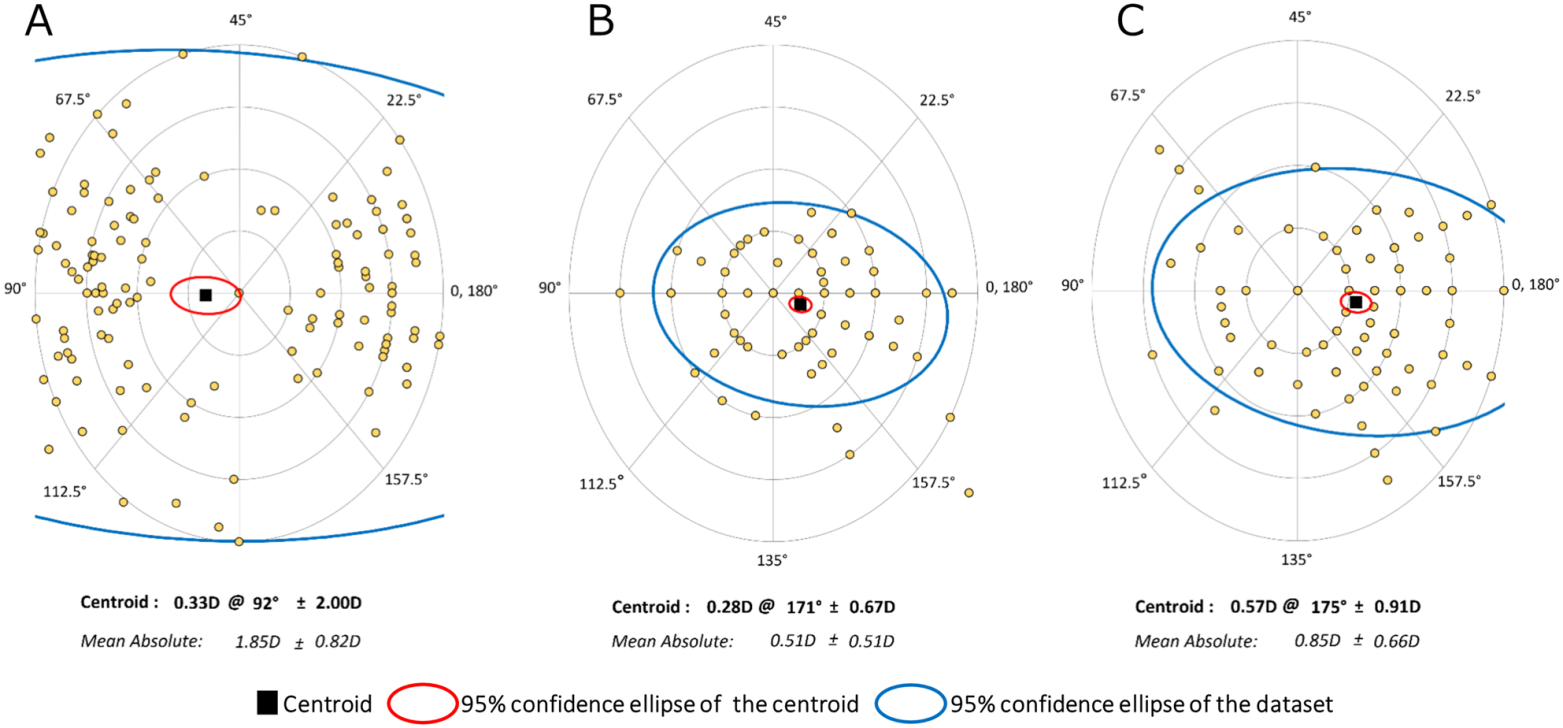
Double-angle plot analyses of preoperative corneal astigmatism (**A**), manifest astigmatism at 1 month postoperatively (**B**), and manifest astigmatism at 8 years postoperatively (**C**). Each ring = 0.5 D.

When postoperative values from 1 month to 8 years were compared, statistically significant changes were found in J_0_ (*p* < 0.001), but not in J_45_ (*p* = 0.743). The mean J_0_ at 5 years (*p* = 0.008) and 8 years (*p* < 0.001) postoperatively were significantly smaller than the mean J_0_ at 1 month postoperatively. From 1 month to 8 years after surgery, J_0_ declined by 0.201 ± 0.563 D. Double-angle plot analyses of preoperative corneal astigmatism and manifest astigmatism at 1 month and 8 years postoperatively are shown in Fig. 2.

**Figure 3:**
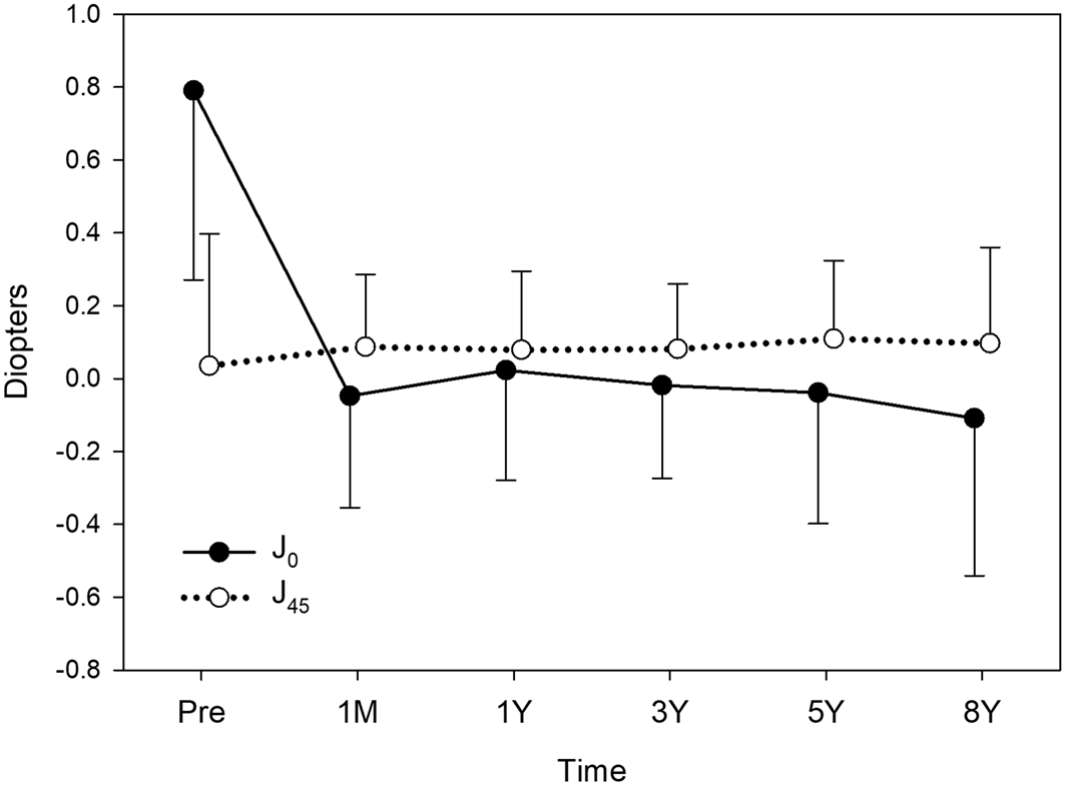
Time course of changes in astigmatism in eyes with preoperative with-the-rule astigmatism. Toric IOL implantation significantly decreased J_0_ (*p* < 0.001), but not J_45_ (*p* = 0.782). Postoperative values from 1 month to 8 years were not significantly different in J_0_ (*p* = 0.511) and J_45_ (*p* = 0.679).

**Figure 4:**
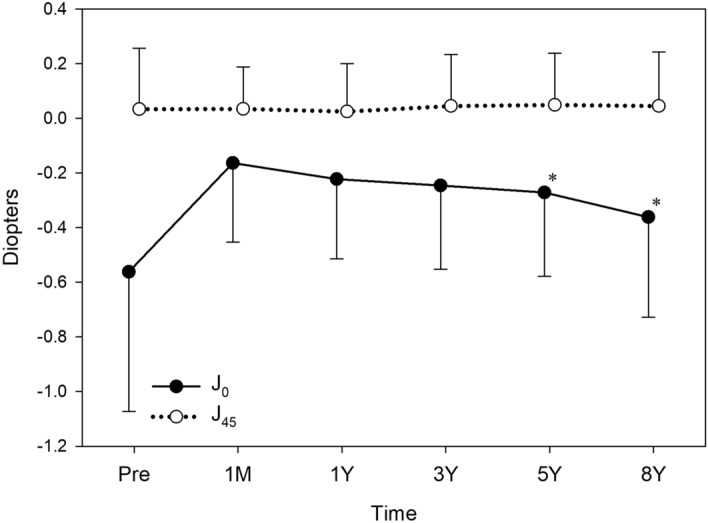
Time course of changes in astigmatism in eyes with preoperative against-the-rule astigmatism. Toric IOL implantation significantly increased J_0_ (*p* < 0.001), but not J_45_ (*p* = 0.825). When postoperative values were compared, J_0_ showed significant changes after surgery (*p* < 0.001), and J_0_ values at 5 years (*p < 0.001) and 8 years (*p < 0.001) were significantly smaller than that at 1 month postoperatively. The mean J_45_ remained stable from 1 month to 8 years after surgery (*p* = 0.964).

**Figure 5:**
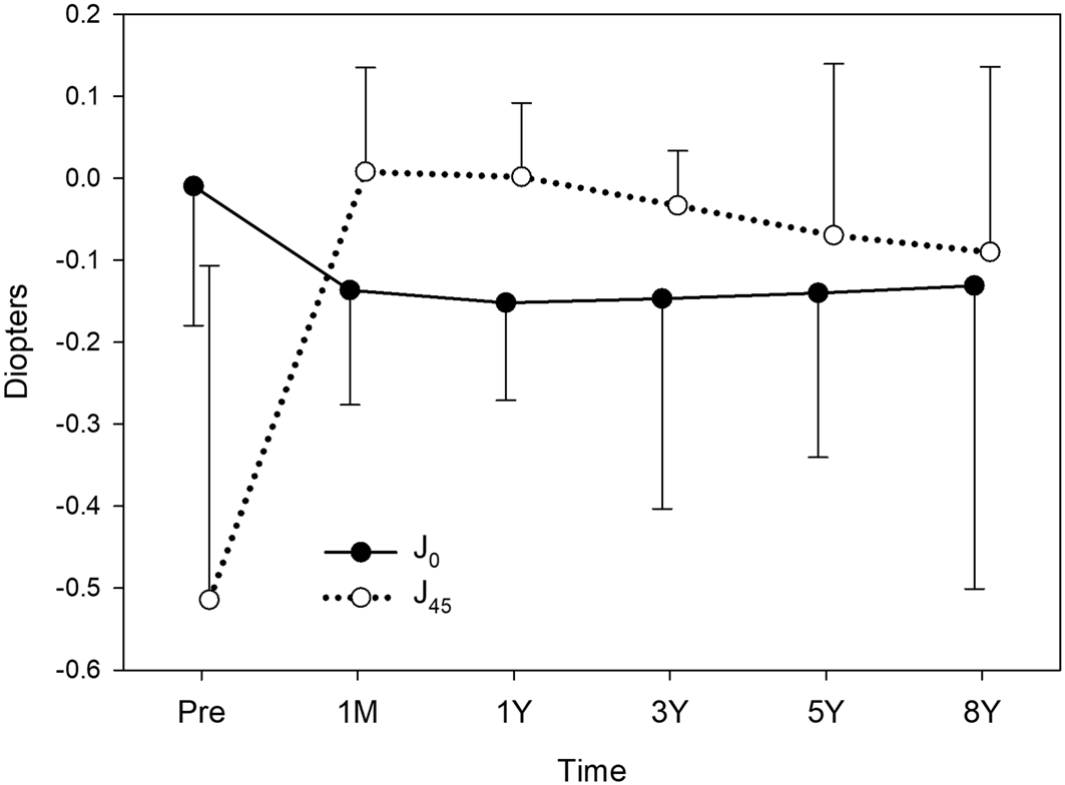
Time course of changes in astigmatism in eyes with preoperative oblique astigmatism. Toric IOL implantation significantly increased J_45_ (*p* = 0.045), but not J_45_ (*p* = 0.912). When postoperative values were compared, J_0_ (*p* = 0.972) and J_45_ (*p* = 0.189) did not change significantly from 1 month to 8 years.

Changes in J_0_ and J_45_ were analysed in eyes with preoperative WTR (Fig. 3), ATR (Fig. 4), and oblique astigmatism (Fig. 5). Toric IOL implantation significantly reduced preoperative astigmatism in all groups. In eyes with WTR astigmatism, surgery significantly decreased J_0_ (*p* < 0.001), but not J_45_ (*p* = 0.782). In eyes with ATR astigmatism, J_0_ significantly increased by surgery (*p* < 0.001), while no change was found in J_45_ (*p* = 0.825). In eyes with oblique astigmatism, surgery induced a significant increase in J_45_ (*p* = 0.045), but not in J_0_ (*p* = 0.912).

When postoperative values from 1 month to 8 years were compared, there were no significant fluctuations in J_0_ (*p* = 0.511) and J_45_ (*p* = 0.679) in eyes with WTR astigmatism (Fig. 3). In eyes with ATR astigmatism (Fig. 4), J_0_ value showed significant changes after surgery (*p* < 0.001), and J_0_ at 5 years (*p* < 0.001) and 8 years (*p* < 0.001) postoperatively were significantly smaller than that at 1 month postoperatively. The mean J_45_ remained steady after surgery (*p* = 0.964). In eyes with oblique astigmatism (Fig. 5), J_0_ (*p* = 0.972) and J_45_ (*p* = 0.189) did not fluctuate significantly from 1 month to 8 years after surgery.

**Figure 6:**
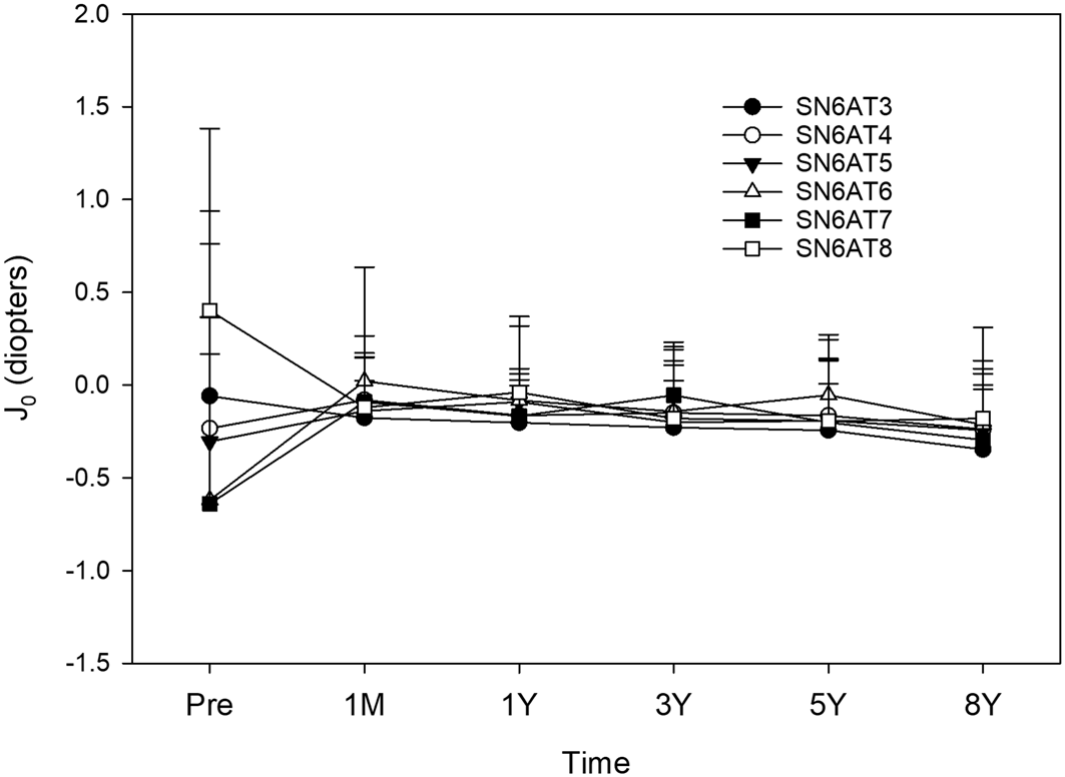
Time course of changes in J_0_ by the model of toric IOLs. Postoperative J_0_ values were not different among the models (*p* > 0.05).

**Figure 7:**
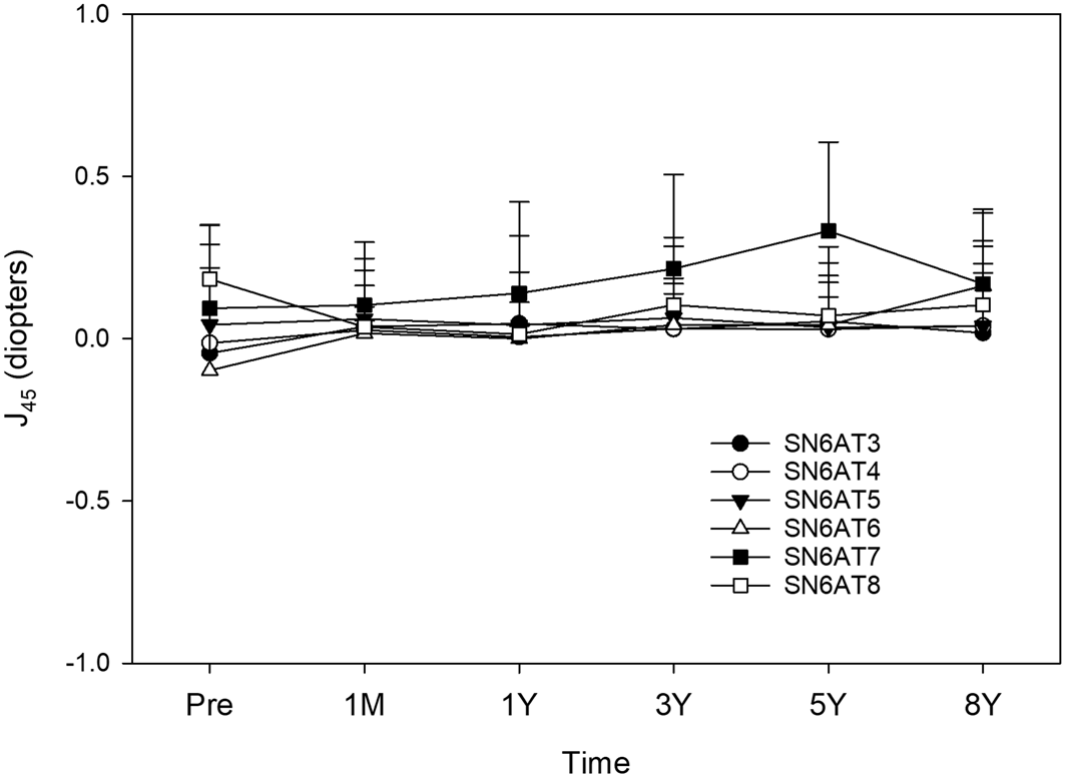
Time course of changes in J_45_ by the model of toric IOLs. Postoperative J_45_ values were not different among the models (*p* > 0.05).

Postoperative time course of changes in J_0_ (Fig. 6) and J_45_ (Fig. 7) was not significantly different among the models of toric IOLs.

### Visual acuity

**Figure 8:**
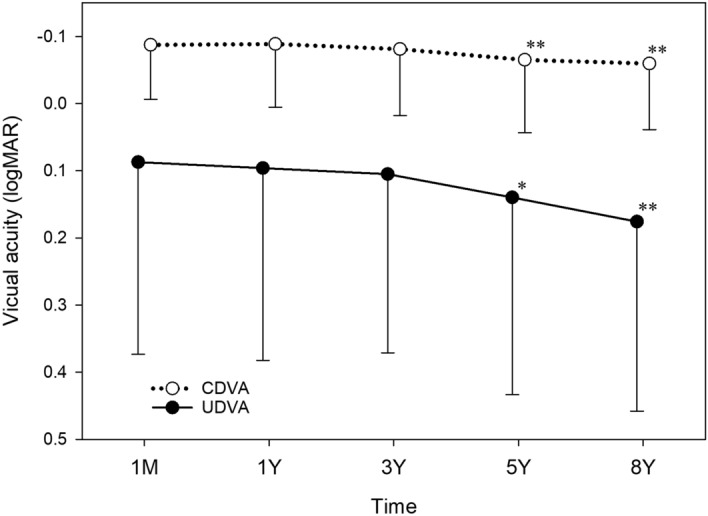
Time course of changes in corrected (CDVA) and uncorrected distance visual acuity (UDVA). Both UDVA (*p* < 0.001) and CDVA (*p* < 0.001) showed significant changes after surgery. UDVA at 5 years (*p = 0.004) and 8 years (**p < 0.001) postoperatively was significantly worse than UDVA at 1 month postoperatively. CDVA at 5 years (**p < 0.001) and 8 years **(*p* < 0.001) after surgery was significantly worse than CDVA at 1 month postoperatively. *logMAR* logarithm of minimum angle of resolution.

The time course of changes in UDVA and CDVA after surgery was analysed. Both UDVA (*p* < 0.001) and CDVA (*p* < 0.001) showed significant changes after surgery (Fig. 8). UDVA at 5 years (*p* = 0.004) and 8 years (*p* < 0.001) postoperatively was significantly worse than UDVA at 1 month postoperatively. CDVA at 5 years (*p* = 0.001) and 8 years (*p* < 0.001) after surgery was significantly worse than CDVA at 1 month postoperatively.

**Figure 9:**
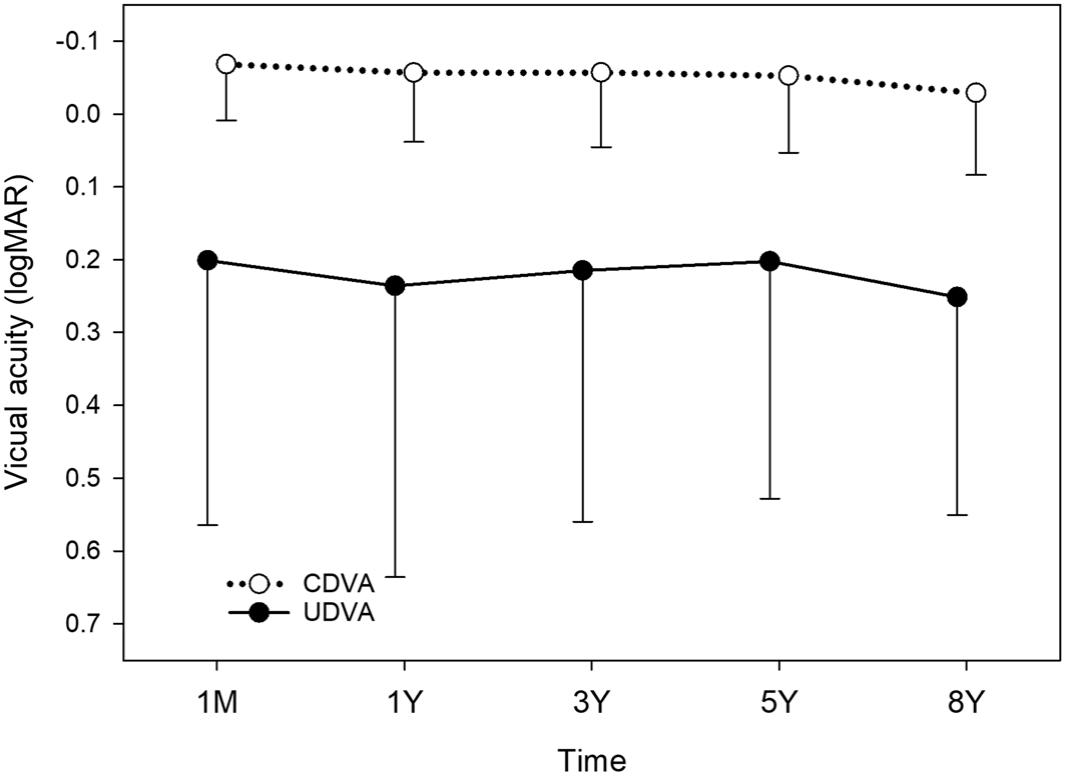
Time course of changes in uncorrected (UDVA) and corrected distance visual acuity (CDVA) in eyes with preoperative with-the-rule astigmatism. There were no significant changes in UDVA (*p* = 0.064) and CDVA (*p* = 0.109) after surgery. *logMAR* logarithm of minimum angle of resolution.

**Figure 10:**
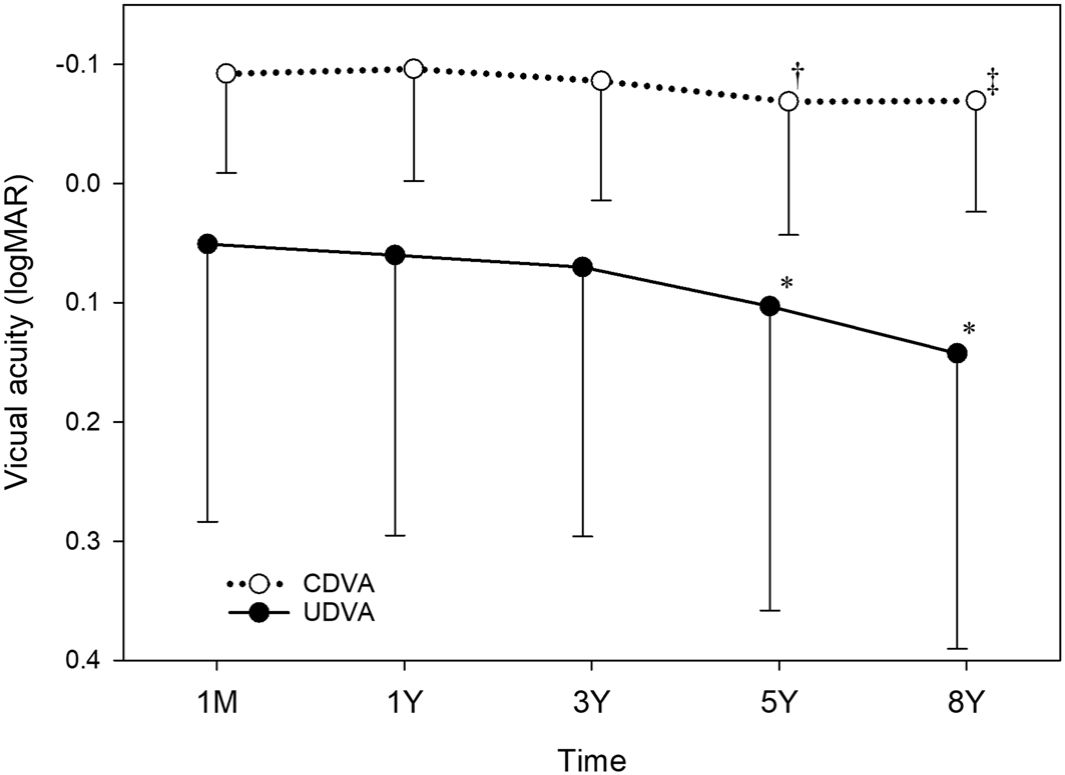
Time course of changes in uncorrected (UDVA) and corrected distance visual acuity (CDVA) in eyes with preoperative against-the-rule astigmatism. UDVA (*p* < 0.001) and CDVA (*p* < 0.001) showed a significant decline after surgery. UDVA at 5 years (*p = 0.009) and 8 years (**p < 0.001) postoperatively was significantly worse than UDVA at 1 month postoperatively. CDVA at 5 years (†*p* = 0.003) and 8 years (‡*p* = 0.002) after surgery was significantly worse than CDVA at 1 month postoperatively. *logMAR* logarithm of minimum angle of resolution.

**Figure 11:**
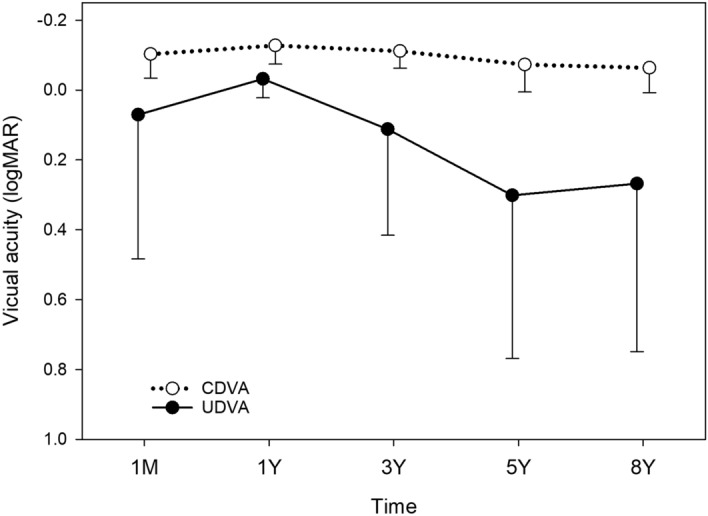
Time course of changes in uncorrected (UDVA) and corrected distance visual acuity (CDVA) in eyes with preoperative oblique astigmatism. There were no significant changes in UDVA (*p* = 0.074) and CDVA (*p* = 0.113). *logMAR* logarithm of minimum angle of resolution.

Visual acuity changes were analysed in eyes with preoperative WTR (Fig. 9), ATR (Fig. 10), and oblique astigmatism (Fig. 11). In eyes with WTR astigmatism, both UDVA (*p* = 0.064) and CDVA (*p* = 0.109) remained stable after surgery. In eyes with oblique astigmatism, postoperative changes in UDVA (*p* = 0.074) and CDVA (*p* = 0.113) were not statistically significant.

In eyes with ATR astigmatism (Fig. 10), UDVA (*p* < 0.001) and CDVA (*p* < 0.001) showed significant declines after surgery. UDVA at 5 years (*p* = 0.009) and 8 years (*p* < 0.001) postoperatively was significantly worse than UDVA at 1 month postoperatively. CDVA at 5 years (*p* = 0.003) and 8 years (*p* = 0.002) after surgery was significantly worse than CDVA at 1 month postoperatively. While UDVA considerably deteriorated from 0.051 logMAR (20/22) at 1 month to 0.143 logMAR (20/28) at 8 years, changes in CDVA were minimum from − 0.092 logMAR (20/16) at 1 month to − 0.069 logMAR (20/17) at 8 years. Changes in logMAR UDVA from 1 month to 8 years (0.188 ± 0.014) were significantly larger than those in logMAR CDVA during the same period (0.077 ± 0.006) (*p* < 0.001).

**Figure 12:**
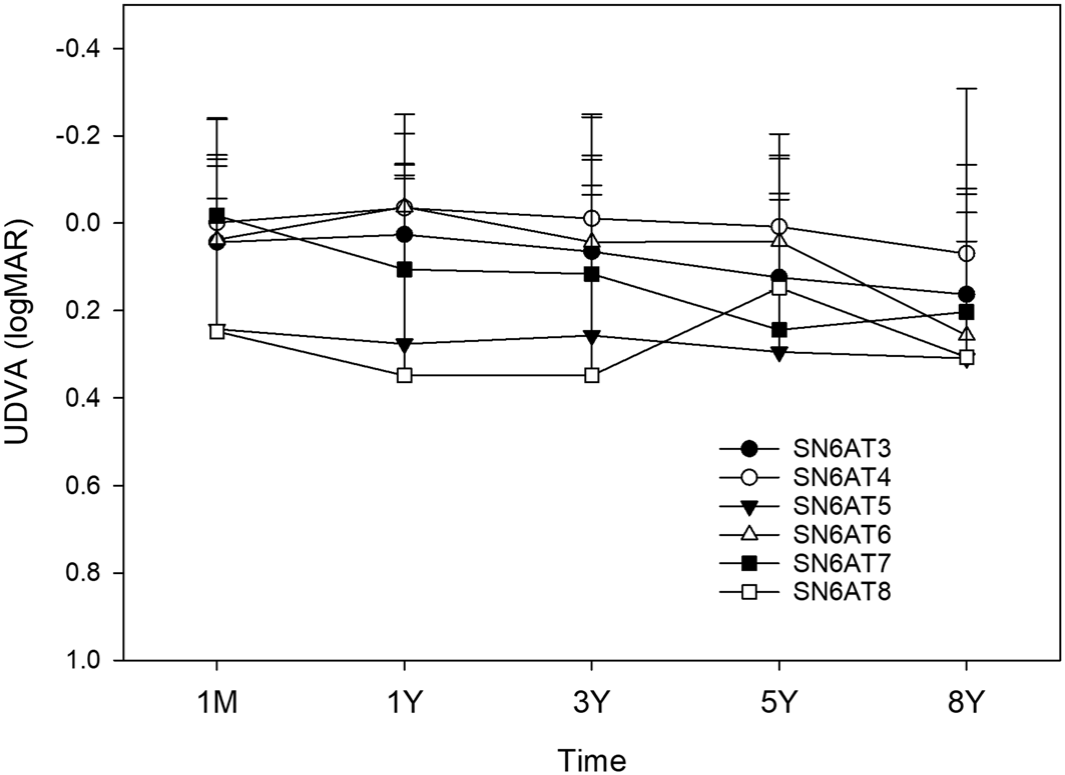
Time course of changes in uncorrected distance visual acuity (UDVA) by the model of toric IOLs. UDVA was not different among the models (*p* > 0.05). *logMAR* logarithm of minimum angle of resolution.

**Figure 13:**
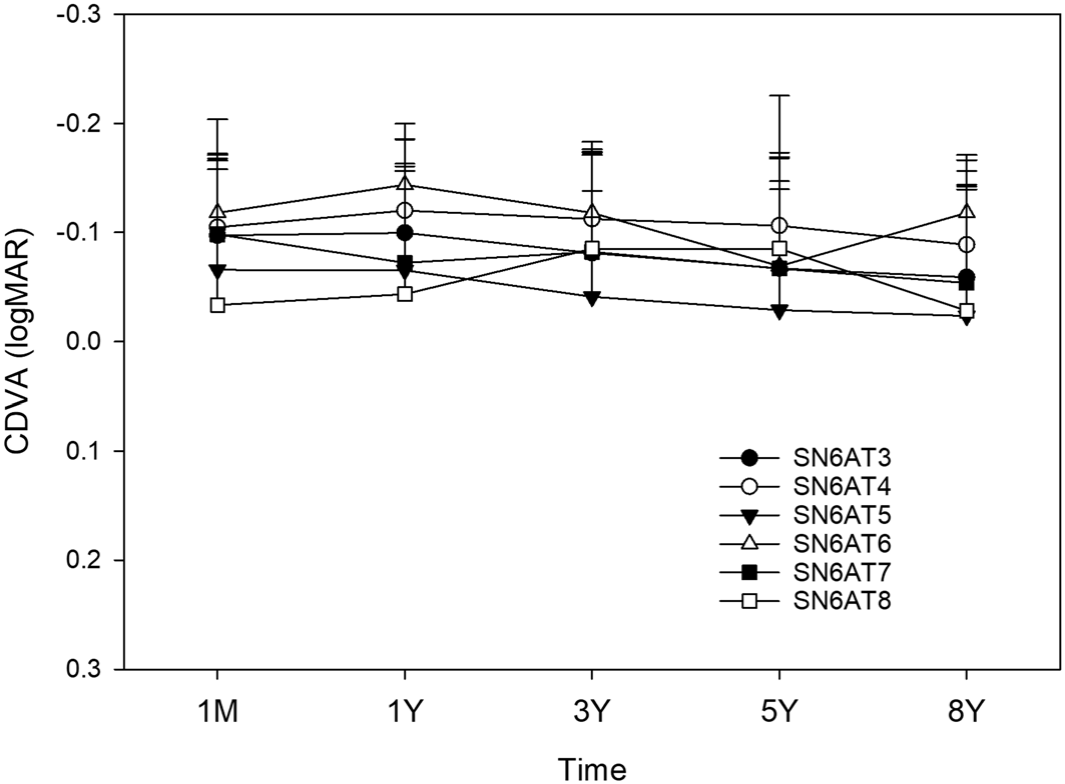
Time course of changes in corrected distance visual acuity (CDVA) by the model of toric IOLs. UDVA was not different among the models (*p* > 0.05). *logMAR* logarithm of minimum angle of resolution.

Postoperative time course of changes in UDVA (Fig. 12) and CDVA (Fig. 13) was not significantly different among the models of toric IOLs.

## Discussion

We found that toric IOL implantation significantly reduced pre-existing astigmatism regardless of the type of preoperative corneal astigmatism; by decreasing power vector notation J_0_ (horizontal/vertical astigmatism component) in eyes with WTR astigmatism, increasing J_0_ in eyes with ATR astigmatism, and correcting J_45_ (oblique astigmatism component) in eyes with oblique astigmatism. Long-term astigmatism-correcting effects of toric IOLs, however, varied depending on the type of preoperative astigmatism. In eyes with WTR and oblique astigmatism, postoperative astigmatism remained stable from 1 month to 8 years after surgery. On the other hand, in eyes with ATR astigmatism, there was a constant decline in postoperative J_0_, and mean J_0_ at 5 years and later was significantly smaller than that at 1 month postoperatively, indicating a significant ATR astigmatic shift after surgery. From 1 month to 8 years postoperatively, J_0_ was reduced by 0.201 ± 0.563 D. This result is in good agreement with a previous study by Hayashi et al.^27^ who reported ATR astigmatic changes of 0.33 D over 10 years after 4.1-mm incision sutureless cataract surgery in patients who were 61.8 ± 6.0 years old at baseline. They also reported that eyes that did not have surgery showed ATR changes of 0.21 D over 10 years from baseline age of 59.9 ± 5.8 years old. Gudmundsdottir et al.^28^ reported a mean ATR astigmatic change of 0.13 D over 5 years in a normal adult population who were 50 years and older at baseline. Judging from the results of these previous and current studies, long-term changes in astigmatism after small incision cataract surgery appear to represent the physiological ATR astigmatic shift that is commonly associated with aging.

In eyes with preoperative WTR and oblique astigmatism, postoperative changes in power vector notation J_0_ and J_45_ were not significant, indicating that astigmatism-correcting effects of toric IOLs in those eyes were maintained at least for 8 years after surgery. Hayashi et al.^24^ assessed long-term changes in the refractive effect of toric IOL implantation and reported similar results. In eyes with preoperative ATR astigmatism, manifest refractive and corneal astigmatism significantly changed toward ATR astigmatism during the 6.6-year follow-up period^24^. In eyes with preoperative WTR astigmatism, manifest refractive and corneal astigmatism did not change significantly for 6.8 years after surgery. Eyes with oblique astigmatism were not included in their study. Longer-term outcomes of toric IOL implantation, however, such as ≥ 10 years remain unknown. In fact, the J_0_ curve in Fig. 3 and J_45_ curve in Fig. 5 in the current study imply that astigmatism-correcting effects of toric IOLs may eventually wane if more extensive studies in terms of both length of the follow-up period and number of subjects are conducted. This would be the subject of future studies.

Until now, long-term time-course of changes in visual acuity after toric IOL implantation have not been well studied. We found that the overall trends of postoperative visual acuity were similar to those of postoperative astigmatic changes aforementioned. In eyes with preoperative WTR and oblique astigmatism, postoperative UDVA and CDVA remained steady from 1 month to 8 years. On the other hand, in eyes with preoperative ATR astigmatism, both UDVA and CDVA significantly deteriorated at 5 years and later after surgery. In particular, changes in UDVA were more prominent than those in CDVA. While UDVA considerably declined from 0.051 logMAR (20/22) at 1 month to 0.143 logMAR (20/28) at 8 years, changes in CDVA were minimum from − 0.092 logMAR (20/16) at 1 month to − 0.069 logMAR (20/17) at 8 years. Changes from 1 month to 8 years were significantly larger in UDVA than in CDVA. From the current results alone, it is impossible to determine the exact mechanism of action underlying deterioration of UDVA and CDVA after surgery. Especially, changes in regular astigmatism cannot explain the worsening of CDVA. We assume that deterioration of UDVA reflects the long-term ATR astigmatic shifts, while changes in CDVA may be attributable to, at least in part, aging changes in visual function including undetected retinal pathologies^29,30^ and increases in irregular corneal astigmatism^31,32^. It has been reported that corneal irregular astigmatism as measured by corneal topography was significantly larger in eyes with ATR astigmatism than in those with WTR astigmatism^33^.

Six models of toric IOLs were used in the current study, SN6AT3–8. The postoperative time course of changes in the power vector notation J_0_ (Fig. 6) and J_45_ (Fig. 7) did not differ significantly among the different models of toric IOL. Similarly, postoperative UDVA (Fig. 12) and CDVA (Fig. 13) were not significantly different among toric IOL models. Thus, all models of toric IOLs used in the current study were almost equally effective to treat cataract patients with pre-existing astigmatism. Xiao et al.^34^ demonstrated that the optical quality after surgery was not significantly different among eyes that received toric IOLs with a different cylinder power, SN60T3 to T6. Bauer et al.^35^ showed comparable surgical outcomes after implantation of SN60T3 to T5. In the current study, we also confirmed that toric IOLs SN6AT3 to AT8 provided comparable and satisfactory long-term clinical outcomes.

There were several limitations to the current study. First, because this study was conducted in a retrospective manner, several important data were missing, such as amount of toric IOL axis misalignment, postoperative keratometry, and corneal topography to evaluate irregular astigmatism. In addition, although eyes with apparent ocular disorders were excluded, the judgement was based only on the retrospective chart review. Second, number of eyes with preoperative oblique astigmatism was small, and thus the statistical analyses in that group may have been underpowered. Similarly, number of eyes with higher toricity IOLs, such as SN6AT6–8, is limited, yielding insufficient statistical power in these subgroups.

In conclusion, long-term surgical outcomes of cataract surgery with toric IOL implantation were investigated. Preoperative corneal and postoperative manifest astigmatism was converted to power vector notations J_0_ (horizontal/vertical component) and J_45_ (oblique component). In eyes with preoperative WTR and oblique astigmatism, the astigmatism-correcting effects continued during the 8-year follow-up period. In eyes with preoperative ATR astigmatism, postoperative astigmatism showed an ATR shift, and J_0_ at 5 and 8 years postoperatively was significantly lower than that at 1 month postoperatively. Visual acuity, especially UDVA, at 5 and 8 years after surgery was significantly worse than that at 1 month after surgery. Given these long-term trends, surgeons may consider overcorrection of astigmatism in eyes with preoperative ATR astigmatism at the time of cataract surgery using toric IOLs.

## Data Availability

The datasets generated during and/or analyzed during the current study are available from the corresponding author on reasonable request.
